# Além do Escore GRACE SCA: É Necessário um Modelo Diferente para Homens e Mulheres após IAMCSST?

**DOI:** 10.36660/abc.20230060

**Published:** 2024-04-17

**Authors:** José Sergio Nascimento Silva, Isly Maria Lucena de Barros, Jorge Augusto Nunes Guimarães, Davide Cao, Sílvia Marinho Martins, Tawanna Xavier Marques de Carvalho, Rayssa Santana de Farias, Viviana Lemke, Roxana Mehran, Rodrigo Pedrosa

**Affiliations:** 1 Universidade de Pernambuco PROCAPE Recife PE Brasil Universidade de Pernambuco – PROCAPE, Recife, PE – Brasil; 2 Hospital Agamenon Magalhães Recife PE Brasil Hospital Agamenon Magalhães, Recife, PE – Brasil; 3 Icahn School of Medicine at Mount Sinai New York EUA Icahn School of Medicine at Mount Sinai, New York – EUA; 4 Real Hospital Português de Beneficência em Pernambuco Recife PE Brasil Real Hospital Português de Beneficência em Pernambuco – Realcor, Recife, PE – Brasil; 5 Cardiocare Clínica Cardiológica Curitiba PR Brasil Cardiocare Clínica Cardiológica, Curitiba, PR – Brasil; 6 Icahn School of Medicine at Mount Sinai Zena Michael A Wiener Cardiovascular Institute New York EUA Icahn School of Medicine at Mount Sinai Zena and Michael A Wiener Cardiovascular Institute, New York – EUA

**Keywords:** Sexo, Mulheres, Síndrome Coronariana Aguda, Intervenção Coronária Percutânea, Doença Arterial Coronariana

## Abstract

**Fundamento:**

As mulheres, em comparação aos homens, apresentam piores resultados após a síndrome coronariana aguda (SCA). No entanto, ainda não está claro se o sexo feminino em si é um preditor independente de tais eventos adversos.

**Objetivo:**

Este estudo tem como objetivo avaliar a associação entre o sexo feminino e a mortalidade hospitalar após infarto do miocárdio com supradesnivelamento do segmento ST (IAMCSST).

**Métodos:**

Conduzimos um estudo de coorte retrospectivo, recrutando pacientes consecutivos com IAMCSST, internados em um hospital terciário de janeiro de 2018 a fevereiro de 2019. Todos os pacientes foram tratados de acordo com as recomendações das diretrizes atuais. Modelos de regressão logística multivariada foram aplicados para avaliar a mortalidade hospitalar utilizando variáveis de GRACE. A precisão do modelo foi avaliada usando o índice c. Um valor de p < 0,05 foi estatisticamente significativo.

**Resultados:**

Dos 1.678 pacientes com SCA, 709 apresentaram IAMCSST. A população era composta por 36% de mulheres e a idade média era de 61 anos. As mulheres tinham maior idade (63,13 anos vs. 60,53 anos, p = 0,011); apresentavam hipertensão (75,1% vs. 62,4%, p = 0,001), diabetes (42,2% vs. 27,8%, p < 0,001) e hiperlipidemia (34,1% vs. 23,9%, p = 0,004) mais frequentemente; e apresentaram menor probabilidade de serem submetidas a intervenção coronária percutânea (ICP) por acesso radial (23,7% vs. 46,1%, p < 0,001). A taxa de mortalidade hospitalar foi significativamente maior em mulheres (13,2% vs. 5,6%, p = 0,001), e o sexo feminino permaneceu em maior risco de mortalidade hospitalar (OR 2,79, IC de 95% 1,15–6,76, p = 0,023). Um modelo multivariado incluindo idade, sexo, pressão arterial sistólica, parada cardíaca e classe de Killip atingiu 94,1% de precisão na previsão de mortalidade hospitalar, e o índice c foi de 0,85 (IC de 95% 0,77–0,93).

**Conclusão:**

Após ajuste para os fatores de risco no modelo de previsão do GRACE, as mulheres continuam em maior risco de mortalidade hospitalar.

## Graphical abstract

**Figura Central:**
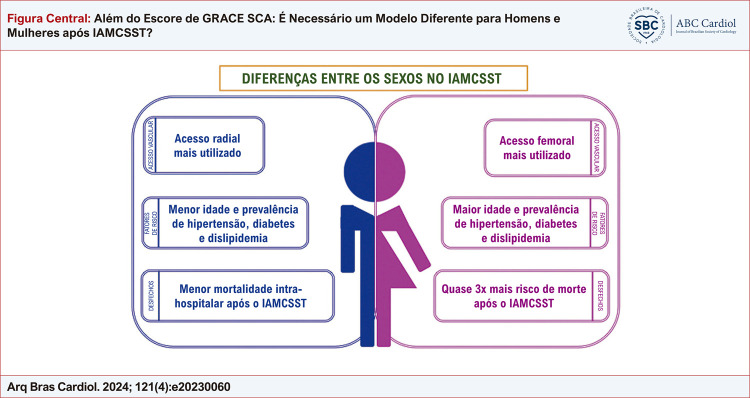
: Além do Escore de GRACE SCA: É Necessário um Modelo Diferente para Homens e Mulheres após IAMCSST?

## Introdução

As mulheres apresentam resultados piores após um infarto agudo do miocárdio com elevação do segmento ST (IAMCSST) em comparação com os homens.^1-5^ Este fenômeno tem sido parcialmente atribuído a fatores como idade avançada na apresentação e maior prevalência de comorbidades.^6-8^ No entanto, ainda não está claro se as diferenças nos resultados relacionadas ao sexo também se devem a uma diferença real na biologia da doença ou são apenas secundárias aos desequilíbrios iniciais e às disparidades relacionadas ao sexo no tratamento e no acesso a cuidados.^9,10^

O escore *Global Registry of Acute Coronary Events* para Síndrome Coronariana Aguda (GRACE SCA) foi proposto pela primeira vez em 2003,^11^ e ainda é recomendado pelas diretrizes mais atuais para prever a mortalidade após SCA.^12,13^ No entanto, apesar do modelo de risco GRACE SCA ser amplamente usado em todo o mundo, ele não considera o sexo um preditor de risco. Além disso, nas últimas duas décadas, diversos aspectos do tratamento da SCA evoluíram e a precisão dos modelos de predição anteriores diminuiu na prática atual.^14^

Sendo assim, a fim de abordar as controvérsias em torno dos piores desfechos observados em mulheres com SCA, procuramos investigar se o sexo feminino está associado a maior risco de mortalidade hospitalar entre pacientes com IAMCSST.

## Métodos

### Desenho do estudo, localização e pacientes

Realizamos um estudo de coorte retrospectivo em um hospital terciário de cardiologia vinculado a um sistema público nacional de saúde, uma referência regional em doenças cardíacas para uma ampla área geográfica e população, que oferece instalações de cateterismo cardíaco de emergência 24 horas e intervenção coronária percutânea (ICP). A população consistiu de pacientes consecutivos maiores de 18 anos e internados com IAMCSST entre janeiro de 2018 e fevereiro de 2019.

### Coleta de dados

Planejamos padronizar todas as variáveis coletadas de acordo com as recomendações do Consórcio Internacional para Medição de Resultados de Saúde (ICHOM).^15^ Os dados foram inseridos em um banco de dados por pessoal dedicado após consulta diária aos prontuários médicos eletrônicos. Todos os pacientes com IAMCSST foram incluídos e seus eletrocardiogramas (ECG) foram revisados por um cardiologista clínico experiente e um cardiologista intervencionista.

### Definições

O diagnóstico de IAMCSST foi considerado se o ECG mostrasse elevação de ST nova ou presumivelmente nova em duas ou mais derivações contíguas ou um novo bloqueio de ramo esquerdo.^16^

O nível de troponina T de alta sensibilidade foi considerado anormal se > 0,014 ng/mL.^16^

Eventos hemorrágicos maiores relacionados à ICP (definidos como uma diminuição de dois pontos na hemoglobina basal ou duas transfusões unitárias de concentrado de glóbulos vermelhos) dentro de 72 horas após a apresentação do IAM, dissecção coronariana, perfuração coronariana, cirurgia de revascularização do miocárdio de emergência para ICP com falha e complicações vasculares que necessitaram de intervenção durante a internação foram consideradas complicações da ICP.^15^

O sucesso da ICP foi definido quando foi obtido fluxo grau 3 do TIMI (trombólise no infarto do miocárdio).^15^

### Aprovação ética

Todos os procedimentos realizados neste estudo estavam de acordo com os padrões éticos do comitê institucional de pesquisa e com a declaração de Helsinque de 1964 e suas alterações posteriores ou padrões éticos equivalentes. O consentimento informado foi obtido de todos os pacientes. O Comitê de Ética local aprovou o protocolo do estudo (número de aprovação: 37458520.0.0000.5192 em 21 de junho de 2017).

### Análise estatística

O tamanho da amostra foi calculado com base em uma frequência de mortalidade hospitalar de 7,5% após IAMCSST, com risco relativo de 1,98^6^ e considerando um erro alfa de 5% com poder do estudo de 80%; e foi considerado necessário um tamanho amostral de 110 pacientes (55 homens e 55 mulheres).

As variáveis contínuas com distribuição não normal foram apresentadas como mediana e intervalo interquartil devido à não normalidade dos dados, enquanto as variáveis categóricas foram apresentadas em números absolutos e percentuais, com os respectivos intervalos de confiança. Os testes de Mann-Whitney e qui-quadrado foram utilizados para comparar as variáveis contínuas e categóricas entre os grupos. A distribuição normal foi avaliada pelo teste de Shapiro-Wilk.

Uma análise de regressão univariada foi realizada e, em seguida, uma regressão logística multivariada por *stepwise*, com seleção retrospectiva para avaliar a associação entre sexo feminino e maior risco de mortalidade hospitalar. As covariáveis candidatas para inclusão no modelo final foram aquelas do escore de risco GRACE SCA (idade, creatinina, frequência/pulso cardíaco, pressão arterial sistólica, parada cardíaca na internação, troponina anormal e classe Killip) e sexo. Os níveis de significância para inclusão ou exclusão das covariáveis do modelo foram de 0,2. A qualidade de ajuste do modelo foi avaliada pelo teste de Hosmer-Lemeshow e o índice c foi obtido. O valor de p foi considerado estatisticamente significativo quando < 0,05. A análise estatística foi realizada utilizando o pacote estatístico IBM SPSS 21.

## Resultados

### Características basais

De um total de 1.678 pacientes admitidos com SCA, 709 (255 mulheres e 454 homens) apresentaram IAMCSST e foram incluídos no estudo. As características basais dos pacientes estão resumidas na Tabela 1. As mulheres tinham maior idade e tinham diabetes, hipertensão e hiperlipidemia com mais frequência do que os homens. Não houve diferenças significativas entre os sexos em relação à raça/etnia/cor da pele, tabagismo, histórico familiar de doença arterial coronariana (DAC), histórico de acidente vascular encefálico ou infarto do miocárdio (IM), doença arterial periférica, doença pulmonar obstrutiva crônica e doença renal crônica.

**Tabela 1:** – Características basais de todos os pacientes da coorte, estratificados por sexo

Variável	Masculino (n = 454)	Feminino (n = 255)	Todos (n = 709)	Valor de p
Idade (mediana, IIQ*, anos)	60 (52 - 69)	62 (54 - 73)	61 (53 - 70)	p = 0,011*
**Raça/etnia/cor da pele (n, %)**				p = 0,238^†^
Brancos	64 (16)	27 (11,9)	91 (14,5)	
Pretos	42 (10,5)	30 (13,3)	72 (11,5)	
Parda	290 (72,3)	163 (72,1)	453 (72,2)	
Amarelo	5 (1,2)	6 (2,7)	11 (1,8)	
**Fatores de risco (n, %)**				
Hipertensão	274 (62,4)	187 (75,1)	461 (67)	p = 0,001^†^
Diabetes	122 (27,8)	105 (42,2)	227 (33)	p < 0,001^†^
Tabagismo	174 (39,6)	103 (41,4)	277 (40,3)	p = 0,657^†^
Hiperlipidemia	105 (23,9)	85 (34,1)	190 (27,6)	p = 0,004^†^
DAC^†^ histórico familiar	129 (29,4)	72 (28,9)	201 (29,2)	p = 0,897^†^
**Histórico (n, %)**				
Infarto do miocárdio	64 (14,6)	29 (11,6)	93 (13,5)	p = 0,280^†^
AVC	29 (6,6)	15 (6,0)	44 (6,4)	p = 0,764^†^
Doença arterial periférica oclusiva	14 (3,2)	9 (3,6)	23 (3,3)	p = 0,765^†^
Doença renal crônica	16 (3,6)	6 (2,4)	22 (3,2)	p = 0,376^†^
ICP^‡^	41 (10,5)	19 (8,8)	60 (9,9)	p = 0,492^†^
CRM^§^	8 (2,0)	4 (1,8)	12 (2,0)	p = 1,000^†^
Diálise	8 (1,8)	5 (2,0)	13 (1,9)	p = 1,000^‡^
**Medicamentos nos últimos 7 dias (n, %)**				
Aspirina	66 (17,2)	41 (19,2)	107 (17,9)	p = 0,529^†^
Estatina	66 (17,2)	35 (16,4)	101 (16,9)	p = 0,814^†^
Clopidogrel	23 (6,0)	8 (3,8)	31 (5,2)	p = 0,239^†^
Insulina	18 (4,7)	22 (10,3)	40 (6,7)	p = 0,008^†^
**Características de apresentação**				
Frequência cardíaca (mediana, IIQ, batimentos/min)	78 (70 - 90)	81,5 (72 - 96)	80 (70 - 92)	p = 0,007*
Pressão arterial sistólica (mediana, IIQ, mmHg)	130 (110 - 150)	120 (110 - 140)	130 (110 - 140)	p = 0,027*
Parada cardíaca (n, %)	19 (4,4)	12 (5,0)	31 (4,6)	p = 0,722^†^
Nível de creatinina (mediana, IIQ, mg/dL)	0,94 (0,80 –1,13)	0,79 (0,65 - 1,06)	0,90 (0,74 - 1,11)	p < 0,001*
**Classificação Killip (n, %)**				p = 0,148^†^
Classe I	286 (81,5)	150 (76,9)	436 (79,0)	
Classe > I	65 (18,5)	45 (23,1)	110 (20,1)	
**Marcador de necrose (mediana, IIQ, ng/ml)**				
Troponina T cardíaca de alta sensibilidade	3,51 (0,87 - 8,93)	2,50 (0,59 - 6,64)	3,20 (0,77 - 8,20)	p = 0,062*
**Localização da elevação do segmento ST (n, %)**				p = 0,645^†^
Anterior	211 (54,7)	108 (49,5)	319 (52,8)	
Inferior	159 (41,2)	94 (43,1)	253 (41,9)	
Lateral	88 (22,8)	59 (27,1)	147 (24,3)	
Posterior	10 (2,6)	6 (2,8)	16 (2,6)	

*IIQ: intervalo interquartil; ^†^DAC: doença arterial coronariana; AVC: acidente vascular cerebral; ^‡^ICP: intervenção coronária percutânea; ^§^CRM: cirurgia de revascularização do miocárdio. *Valores de p se referem ao teste de Mann-Whitney. ^†^Os valores de p referem-se ao teste qui-quadrado de Pearson. ^‡^ Valores de p referem-se ao teste Exato de Fisher.

### Variáveis e condições pré-hospitalares

Antes da internação na instituição terciária, a maioria dos pacientes havia recebido atendimento em hospital de emergência geral, dos quais 61,4% foram tratados principalmente dentro de três horas desde o início da dor torácica. Comparativamente, 45,9% foram encaminhados para cuidados terciários após cinco horas do início dos sintomas e 24% após mais de dez horas. No entanto, quando avaliados os tempos do início dos sintomas até o atendimento hospitalar (tempo dor-porta), até a aquisição do ECG (tempo dor-ECG) e da internação hospitalar terciária até a ICP (tempo porta-balão), constatou-se que esses períodos não foram significativamente diferentes em relação ao sexo. Entretanto, observamos um tempo total de isquemia diferente (tempo entre início dos sintomas e o balão), mostrando que os homens foram submetidos à ICP primária mais rapidamente que as mulheres (Tabela 2).

**Tabela 2:** – Diferenças e semelhanças entre os sexos quanto ao tratamento clínico e invasivo

Variável	Masculino	Feminino	Todos	Valor de p

	n (%)	n (%)	n (%)	
**Cenário clínico**				
Tempo total de isquemia (mediana, IIQ*, horas)	13,86 (8,81 - 17,00)	18,00 (10,5 - 20,33)	14,77 (9,30 - 19,68)	p<0,001*
**Tempo dor-porta (horas)**				p=0,416*
Até 2	64 (17,6)	28 (13,7)	92 (16,2)	
2–5	100 (27,5)	50 (24,5)	150 (26,5)	
5–10	96 (26,4)	59 (28,9)	155 (27,3)	
> 10	103 (28,4)	67 (32,8)	170 (30)	
**Tempo dor-ECG^**†**^ (horas)**				p=0,265*
< 1	117 (37,4)	45 (29,8)	162 (34,9)	
1–3	92 (29,4)	57 (37,7)	149 (32,1)	
3–10	68 (21,7)	33 (21,9)	101 (21,8)	
> 10	36 (11,5)	16 (10,6)	52 (11,2)	
**Tempo porta-balão (horas)**				p=0,174*
< 1	59 (31,1)	27 (23,1)	86 (28,0)	
1–4	58 (30,5)	33 (28,2)	91 (29,6)	
4–10	42 (22,1)	27 (23,1)	69 (22,5)	
> 10	31 (16,3)	30 (25,6)	61 (19,9)	
Terapia fibrinolítica	17 (4,0)	6 (2,5)	23 (3,4)	p=0,304^†^
Uso de inibidor de glicoproteína IIb/IIIa	27 (5,9)	14 (5,5)	41 (5,8)	p=0,802^†^
IM espontâneo^‡^	372 (96,1)	209 (97,7)	581 (96,7)	p=0,836^‡^
IM relacionado à trombose de stent^‡^	10 (2,6)	3 (1,4)	13 (2,2)	p=0,836^‡^
**Achados relacionados ao cateterismo cardíaco**				p=0,515^†^
Envolvimento uniarterial	172 (41,1)	84 (36,5)	256 (39,5)	
Envolvimento bi ou triarterial	104 (54,1)	130 (56,5)	356 (55,0)	
Envolvimento de doença principal esquerda	24 (5,8)	14 (6,3)	38 (6,0)	
Nenhuma obstrução significativa	20 (4,8)	16 (7,0)	36 (5,6)	
**Detalhes do ICP^**§**^**				
Acesso radial	155 (46,1)	45 (23,7)	200 (38)	p<0,001^‡^
1 ou 2 stents implantados	309 (91,4)	176 (91,2)	485 (91,3)	p=0,996^†^
Stent convencional implantado	302 (92,9)	172 (92,0)	474 (92,6)	p=0,849^‡^
Taxa de procedimento de sucesso	311 (96,0)	176 (94,1)	487 (95,3)	p=0,336^†^
TIM ^//^ 3 fluxo final	254 (89,4)	146 (87,4)	400 (88,7)	p=0,671^‡^
Principais complicações hemorrágicas	5 (1,6)	6 (3,3)	11 (2,2)	p=0,344^‡^
Indicação para CRM^¶^	9 (2,7)	6 (3,3)	15 (2,9)	p=0,705^†^
Mortalidade geral intra-hospitalar	22 (5,6)	29 (13,2)	51 (8,3)	p=0,001^‡^

*IIQ: intervalo interquartil; ^†^ECG: eletrocardiograma; ^‡^IM: infarto do miocárdio; ^§^ICP: intervenção coronária percutânea; ^//^TIM: trombólise no infarto do miocárdio; ^¶^CRM: cirurgia de revascularização do miocárdio. ^*^Os valores de p referem-se ao teste de Mann-Whitney. ^†^Os valores de p referem-se ao teste qui-quadrado de Pearson. ^‡^Os valores de p referem-se ao teste Exato de Fisher.

### Avaliação clínica e eletrocardiograma

A maioria dos pacientes apresentou IM espontâneo. Na internação hospitalar, as variáveis de dor torácica, choque cardiogênico, parada cardíaca, classe Killip e indicação de tratamento invasivo não foram diferentes entre mulheres e homens. Cerca de um terço dos pacientes realizou um ECG de 12 derivações na primeira hora após o início dos sintomas e a maioria dos indivíduos recebeu um diagnóstico de ECG dentro de três horas, sem diferenças significativas entre os sexos.

Ritmo sinusal e supradesnivelamento do segmento ST da parede anterior, seguido de supradesnivelamento do segmento ST da parede inferior, foram as apresentações mais comuns e semelhantes entre os sexos, enquanto o supradesnivelamento do segmento ST isolado em derivação vetorial aumentada direita (aVR) e bloqueio completo de ramo esquerdo ou direito foram incomuns.

### Cateterismo cardíaco e ICP primária

Cateterismo cardíaco de emergência seguido de ICP primária foram realizados na maioria dos pacientes. Alguns pacientes foram submetidos a ICP eletiva e uma minoria não teve indicação de estratégia invasiva devido a complicações clínicas não cardíacas, como hemorragia gástrica ou fragilidade, especialmente em pacientes muito idosos, a critério do médico responsável. DAC não obstrutiva foi encontrada em 36 pacientes, independentemente do sexo.

O acometimento da doença uni, bi ou multiarterial e do tronco da cornária esquerda não diferiu entre os sexos. O acesso radial teve maior probabilidade de ser realizado em homens em comparação com mulheres. O número e tipo de *stent* (não-farmacológico ou farmacológico), o sucesso da ICP e o fluxo final TIMI (Trombólise no Infarto do Miocárdio) não foram diferentes entre os grupos.

A grande maioria dos pacientes recebeu um *stent* não-farmacológico. Qualquer complicação relacionada à ICP ocorreu em um pequeno número de pacientes e foi causada principalmente por sangramento. Níveis basais de creatinina e pressão arterial sistólica foram mais baixos nas mulheres do que nos homens, enquanto a frequência cardíaca foi mais alta nas mulheres do que nos homens.

### Mortalidade hospitalar

**Figura 1:**
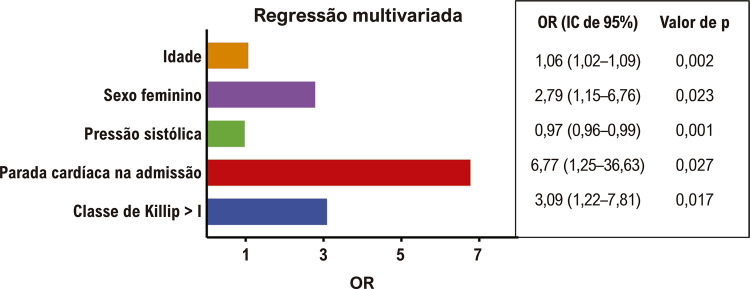
– Modelo multivariado final para mortalidade hospitalar após infarto agudo do miocárdio com supradesnivelamento do segmento ST.

Cinquenta e um pacientes morreram. Mulheres tiveram uma taxa de mortalidade significativamente maior, em comparação com os homens. Os resultados do modelo de regressão logística multivariada são apresentados na Tabela 3. Idade, parada cardíaca na internação, classe de Killip > I e sexo feminino foram preditores independentes de mortalidade hospitalar. O modelo final (Figura 1) teve 94,1% de precisão (c^2^ = 12,45, p = 0,132) na previsão da mortalidade hospitalar geral com um bom ajuste do modelo, e o índice c foi de 0,85, com IC de 95% 0,77–0,93.

**Tabela 3:** – Regressões logísticas univariadas e multivariadas para predição de mortalidade hospitalar

Variável	Regressão univariada		Regressão multivariada	
	
	OR^*^ (IC de 95%)	Valor de p	OR^*^ (IC de 95%^**†**^)	Valor de p
Idade (por incremento de 1 ano)	1,06 (1,04–1,09)	< 0,001	1,06 (1,02–1,09)	0,002
Sexo feminino	2,66 (1,48–4,65)	0,001	2,79 (1,15–6,76)	0,023
Nível de troponina T	1,03 (0,99–1,07)	0,185	1,05 (0,99–1,11)	0,143
Nível inicial de creatinina	1,17 (1,00–1,14)	0,047	1,48 (0,96–2,20)	0,079
Frequência cardíaca/pulso	1,02 (1,00–1,04)	0,002	1,02 (1,00–1,04)	0,055
Pressão sistólica	0,97 (0,96–0,98)	< 0,001	0,97 (0,96–0,99)	0,001
Parada cardíaca na internação	10,45 (4,10–26,58)	< 0,001	6,77 (1,25–36,63)	0,027
Classe de Killip > I	4,74 (2,58–8,71)	< 0,001	3,09 (1,22–7,81)	0,017

O modelo logístico final foi estatisticamente significativo e a estatística qui-quadrado de Hosmer-Lemeshow (uma medida da qualidade de ajuste) foi c^2^ = 12,45, com valor de p de 0,132. ^*^OR: razão de chances; ^†^IC: intervalo de confiança.

## Discussão

Até o momento, o presente estudo é a maior coorte proposta especificamente para avaliar o impacto das diferenças entre sexos após IAMCSST no Brasil. Além disso, mostramos que o sexo feminino permaneceu em maior risco de mortalidade hospitalar após IAMCSST, em contraste com o escore de risco GRACE SCA, no qual o sexo não é um preditor. Apesar de as mulheres terem maior idade que os homens em nosso estudo, o sexo feminino permaneceu associado à sobrevida prejudicada mesmo após ajuste multivariável (Figura Central), que incluiu a idade em linha com os achados do estudo de-Miguel-Balsa E et al. Ao validar o GRACE, o sexo feminino foi considerado preditor independente de mortalidade hospitalar na subpopulação com IAMCSST e o sexo não melhorou substancialmente a capacidade discriminativa do escore de GRACE.^16^

Além disso, embora as mulheres apresentassem maior prevalência de comorbidades, como hipertensão, diabetes e dislipidemia, o GRACE SCA não as considera na predição de mortalidade após SCA.^11,16^ Ainda assim, o índice de comorbidade de Charlson foi semelhante em ambos os sexos.^17^ Além disso, outros importantes fatores de risco cardiovascular (tabagismo, histórico familiar de DAC, acidente vascular encefálico ou infarto do miocárdio prévio, doença arterial periférica e doença renal crônica), que demonstraram maior impacto na mortalidade mais elevada, não foram significativamente diferentes entre homens e mulheres.^4,6^

Ademais, sugeriu-se que sintomas atípicos poderiam atrasar o tratamento da SCA e levar a um manejo menos invasivo em mulheres em comparação aos homens.^18^ Alinhado ao tempo total de isquemia diferente encontrado em nosso estudo, um período de espera mais longo para ICP primária é desfavorável para mulheres. Mesmo não sendo parte do modelo de predição de mortalidade GRACE, isso pode ter contribuído para uma maior mortalidade entre as mulheres deste estudo. No entanto, não encontramos diferenças significativas nos tempos dor-porta, dor-ECG e dor-balão, ou na taxa de indicação de ICP entre os sexos.

Em relação ao acesso para cateterismo cardíaco e procedimentos de ICP, nosso estudo mostrou uma diferença significativa na escolha do acesso arterial, sendo o acesso radial menos frequente em mulheres. Taxas de mortalidade mais altas podem estar relacionadas ao aumento do uso de acesso femoral para ICP de SCA entre mulheres devido a hemorragias mais significativas.^19,20^ No entanto, complicações hemorrágicas foram incomuns em nosso estudo e não encontramos qualquer diferença com base no sexo. Além disso, o IAMCSST foi a apresentação mais comum em nossa coorte de estudo e geralmente está associado a piores resultados em comparação com outras apresentações de SCA. No entanto, a localização do IM não foi diferente entre homens e mulheres.

Além disso, embora a ICP primária tenha sido indicada para 85,6% dos pacientes, um número considerável de pacientes com DAC não obstrutiva, indicação de cirurgia de revascularização do miocárdio (CRM) e fase subaguda de IAMCSST teve contraindicação de ICP, independentemente do sexo.

É amplamente reconhecido que mulheres são menos frequentemente diagnosticadas e tratadas após SCA.^18,21^ No entanto, isto não foi demonstrado no nosso estudo, uma vez que mulheres e homens tiveram prescrições de medicamentos, indicações de ICP e taxas de sucesso semelhantes. Embora a percentagem de *stents* não-farmacológicos fosse elevada quando se comparavam os relatórios atuais, esta era independente do sexo e refletia o financiamento limitado do sistema nacional de saúde pública.

### Mortalidade e um modelo multivariado

A taxa de mortalidade hospitalar aumentada observada em mulheres está de acordo com relatos anteriores.^1,2^ Esse achado tem sido atribuído à idade avançada e à maior carga de comorbidades das mulheres. Entretanto, vários estudos mostraram aumento da mortalidade em mulheres mesmo após ajuste para esses fatores de confusão.^22-26^ Sendo assim, após adicionar o sexo feminino ao escore GRACE SCA estabeleceu que variáveis como idade, parada cardíaca na internação e classe de Killip > I permaneceram preditores independentes de mortalidade hospitalar. Por outro lado, a troponina, o nível de creatinina e a frequência cardíaca anormais foram excluídos do modelo.

### Limitações

As limitações deste estudo estão relacionadas ao desenho observacional. Não podemos descartar o viés de seleção e todos os fatores de confusão residuais e/ou não mensurados. Além disso, a amostra é derivada de uma experiência unicêntrica, que analisa apenas a mortalidade intra-hospitalar, podendo subestimar a prevalência de determinados fatores de risco cardiovascular, podendo ter ocorrido classificação incorreta de diagnóstico. Para esta análise, foram considerados apenas pacientes internados, e a possibilidade de maiores taxas de mortalidade após IAMCSST em homens do que em mulheres antes da internação hospitalar pode ter sido um viés de seleção. Em relação às medidas relacionadas à possível influência do sexo sobre o tratamento, acreditamos que, ao incluirmos todos os pacientes consecutivos e realizarmos a coleta de dados de forma intra-hospitalar após o tratamento padrão do IAMCSST, minimizamos esse viés, embora ele não tenha sido excluído. Além disso, o poder estatístico limitado do estudo pode ter afetado a seleção de variáveis em nosso modelo multivariado.

### Implicações para a prática e/ou política

Este achado destaca as disparidades de resultados após SCA em mulheres, em comparação com homens, e deve ser um aspecto considerado na implementação de políticas de saúde para minimizar a mortalidade de mulheres após SCA. Os profissionais clínicos devem ser constantemente treinados para observar particularidades relacionadas ao sexo em cenários clínicos, tais como descrições de sintomas, fatores de risco e evolução clínica após IAMCSST.

## Conclusões

Concluindo, o sexo feminino, adicionado ao escore GRACE, permaneceu associado a maior risco de mortalidade hospitalar após IAMCSST. Além disso, as diretrizes de tratamento recomendadas e seguidas para todos os pacientes com IAMCSST neste estudo podem não ser apropriadas para mulheres. Ainda, o sexo feminino deve ser reconsiderado em futuras iterações da pontuação de risco de mortalidade.
