# Supplementary material for: Além do Escore GRACE SCA: É Necessário um Modelo Diferente para Homens e Mulheres após IAMCSST?
Source: Arq Bras Cardiol. 2024 Apr 17;121(4):e20230060. [Article in Portuguese] doi: 10.36660/abc.20230060 (PMC11098573; doi:10.36660/abc.20230060)
Supplement: Translation

# Translation: Beyond the GRACE ACS Score: Do We Need a Different Model for Men and Women after STEMI?

**DOI:** 10.36660/abc.20230060i

**Authors:** José Sergio Nascimento Silva, Isly Maria Lucena de Barros, Jorge Augusto Nunes Guimarães, Davide Cao, Sílvia Marinho Martins, Tawanna Xavier Marques de Carvalho, Rayssa Santana de Farias, Viviana Lemke, Roxana Mehran, Rodrigo Pedrosa

**Affiliations:** 1 Brazil Universidade de Pernambuco – PROCAPE, Recife, PE – Brazil; 2 Brazil Hospital Agamenon Magalhães, Recife, PE – Brazil; 3 USA Icahn School of Medicine at Mount Sinai, New York – USA; 4 Brazil Real Hospital Português de Beneficência em Pernambuco – Realcor, Recife, PE – Brazil; 5 Brazil Cardiocare Clínica Cardiológica, Curitiba, PR – Brazil; 6 USA Icahn School of Medicine at Mount Sinai Zena and Michael A Wiener Cardiovascular Institute, New York – USA

**Keywords:** Sex, Women, Acute Coronary Syndrome, Percutaneous Coronary Intervention, Coronary Artery Disease

## Abstract

**Background:**

Women, in comparison to men, experience worse outcomes after acute coronary syndrome (ACS). However, whether the female sex per se is an independent predictor of such adverse events remains unclear.

**Objective:**

This study aims to assess the association between the female sex and in-hospital mortality after ST-elevation myocardial infarction (STEMI).

**Methods:**

We conducted a retrospective cohort study by enrolling consecutive STEMI patients admitted to a tertiary hospital from January 2018 to February 2019. All patients were treated per current guideline recommendations. Multivariable logistic regression models were applied to evaluate in-hospital mortality using GRACE variables. Model accuracy was evaluated using c-index. A p-value < 0.05 was statistically significant.

**Results:**

Out of the 1678 ACS patients, 709 presented with STEMI. The population consisted of 36% women, and the median age was 61 years. Women were older (63.13 years vs. 60.53 years, p = 0.011); more often presented with hypertension (75.1% vs. 62.4%, p = 0.001), diabetes (42.2% vs. 27.8%, p < 0.001), and hyperlipidemia (34.1% vs. 23.9%, p = 0.004); and were less likely to undergo percutaneous coronary intervention (PCI) via radial access (23.7% vs. 46.1%, p < 0.001). In-hospital mortality rate was significantly higher in women (13.2% vs. 5.6%, p = 0.001), and the female sex remained at higher risk for in-hospital mortality (OR 2.79, 95% CI 1.15–6.76, p = 0.023). A multivariate model including age, sex, systolic blood pressure, cardiac arrest, and Killip class was 94.1% accurate in predicting in-hospital mortality, and the c-index was 0.85 (95% CI 0.77–0.93).

**Conclusion:**

After adjusting for the risk factors in the GRACE prediction model, women remain at higher risk for in-hospital mortality.

## Introduction

Women have been shown to have poorer outcomes after ST-elevation myocardial infarction (STEMI) compared to men.^1-5^ This phenomenon has been partly attributed to factors such as older age at presentation and higher prevalence of comorbidities.^6-8^ Yet, it remains unclear whether sex-related differences in outcomes are also due to an actual difference in the biology of the disease or only secondary to baseline imbalances and sex-related disparities in treatment and access to care.^9,10^

The Global Registry of Acute Coronary Events for Acute Coronary Syndrome (GRACE ACS) score was first proposed in 2003,^11^ and is still recommended by current guidelines for predicting mortality after ACS.^12,13^ However, despite the GRACE ACS risk model being widely used around the world, it does not consider sex a risk predictor. Furthermore, over the last two decades, several aspects of ACS treatment have evolved, and the accuracy of previous prediction models has decreased in current practice.^14^

Therefore, in order to address the controversies surrounding the worse outcomes experienced by women with ACS, we sought to investigate whether the female sex is associated with a higher risk for in-hospital mortality among STEMI patients.

## Methods

### Study design, location, and patients

We conducted a retrospective cohort study in a tertiary cardiology hospital of a national public healthcare system, which serves as a regional heart disease reference for a large geographical area and population, with 24-hour emergency cardiac catheterization and percutaneous coronary intervention (PCI) facilities. The population consisted of consecutive patients older than 18 years and admitted with STEMI between January 2018 and February 2019.

### Data collection

We planned to standardize all collected variables according to the recommendations of the International Consortium for Health Outcomes Measurement (ICHOM).^15^ Data were entered into a prospective database by dedicated personnel after consulting electronic medical records on a daily basis. All patients with STEMI were enrolled and their electrocardiograms (ECG) were reviewed by an experienced clinical cardiologist and an interventional cardiologist.

### Definitions

The diagnosis of STEMI was considered if the ECG showed new or presumably new ST-elevation in two or more contiguous leads or a new left bundle-branch block.^16^

High-sensitivity troponin T level was considered abnormal at > 0.014 ng/mL.^16^

Major bleeding events related to PCI (defined as a decrease of two points in basal hemoglobin or two packed red blood cells unit transfusions) within 72 hours of index presentation, coronary dissection, coronary perforation, emergent coronary artery bypass grafting for failed PCI, and vascular complications requiring intervention during hospitalization were considered PCI complications.^15^

PCI success was defined when thrombolysis in myocardial infarction (TIMI) grade flow 3 was obtained.^15^

### Ethical approval

All procedures performed in this study were in accordance with the ethical standards of the institutional research committee, and the 1964 Declaration of Helsinki and its later amendments or comparable ethical standards. Informed consent was obtained from all patients. The local Ethics Committee approved the study protocol (approval number: 37458520.0.0000.5192 on June 21, 2017).

### Statistical analysis

Sample size was calculated based on a 7.5% in-hospital mortality frequency after STEMI with a 1.98 relative risk,^6^ considering a 5% alfa error and 80% study power; and a sample size of 110 patients was found necessary (55 men and 55 women).

Continuous variables with non-normal distribution were presented as median and interquartile range due to the non-normality of the data, while categorical variables were presented by absolute numbers and percentages, with the relevant confidence intervals. The Mann-Whitney and chi-squared tests were used to compare the continuous and categorical variables between the groups. Normal distribution was assessed using the Shapiro-Wilk test.

A univariate regression analysis was performed, and then a multivariable stepwise logistic regression with backward selection was used to evaluate the association between the female sex and higher risk for in-hospital mortality. Candidate covariates for inclusion into the final model were those from the GRACE ACS risk score (age, creatinine, heart rate/pulse, systolic blood pressure, cardiac arrest at admission, abnormal troponin, and Killip class) and sex. The significance levels for inclusion or exclusion of the covariates from the model were 0.2. The model’s goodness-of-fit was assessed with the Hosmer-Lemeshow test, and the c-index was obtained. The P-value was considered statistically significant when < 0,05. Statistical analysis was performed using the IBM SPSS 21 statistical package.

## Results

### Baseline characteristics

From a total of 1,678 patients admitted with ACS, 709 (255 women and 454 men) presented with STEMI and were enrolled in the study. The baseline characteristics of the patients are summarized in Table 1. Women were older and had diabetes, hypertension, and hyperlipidemia more often than men. There were no significant differences between the sexes with respect to race/ethnicity/skin color, smoking status, coronary artery disease (CAD) family history, history of stroke or myocardial infarction (MI), peripheral artery disease, chronic obstructive pulmonary disease, and chronic kidney disease.

**Table 1:** – Baseline characteristics of all cohort patients stratified by sex

Variable	Male (n = 454)	Female (n = 255)	All (n = 709)	p Value
Age (median, IQR*, years)	60 (52 - 69)	62 (54 - 73)	61 (53 - 70)	p = 0.011*
**Race/ethnicity/skin color (n, %)**				p = 0.238^†^
Caucasian	64 (16)	27 (11.9)	91 (14.5)	
Black	42 (10.5)	30 (13.3)	72 (11.5)	
Brown	290 (72.3)	163 (72.1)	453 (72.2)	
Yellow	5 (1.2)	6 (2.7)	11 (1.8)	
**Risk factors (n, %)**				
Hypertension	274 (62.4)	187 (75.1)	461 (67)	p = 0.001^†^
Diabetes	122 (27.8)	105 (42.2)	227 (33)	p < 0.001^†^
Smoking	174 (39.6)	103 (41.4)	277 (40.3)	p = 0.657^†^
Hyperlipidemia	105 (23.9)	85 (34.1)	190 (27.6)	p = 0.004^†^
CAD^†^ family history	129 (29.4)	72 (28.9)	201 (29.2)	p = 0.897^†^
**Previous history (n, %)**				
Myocardial infarction	64 (14.6)	29 (11.6)	93 (13.5)	p = 0.280^†^
Stroke	29 (6.6)	15 (6.0)	44 (6.4)	p = 0.764^†^
Occlusive peripheral arterial disease	14 (3.2)	9 (3.6)	23 (3.3)	p = 0.765^†^
Chronic kidney disease	16 (3.6)	6 (2.4)	22 (3.2)	p = 0.376^†^
PCI^‡^	41 (10.5)	19 (8.8)	60 (9.9)	p = 0.492^†^
CABG^§^	8 (2.0)	4 (1.8)	12 (2.0)	p = 1.000^†^
Dialysis	8 (1.8)	5 (2.0)	13 (1.9)	p = 1.000^‡^
**Medications in last 7 days (n, %)**				
Aspirin	66 (17.2)	41 (19.2)	107 (17.9)	p = 0.529^†^
Statin	66 (17.2)	35 (16.4)	101 (16.9)	p = 0.814^†^
Clopidogrel	23 (6.0)	8 (3.8)	31 (5.2)	p = 0.239^†^
Insulin	18 (4.7)	22 (10.3)	40 (6.7)	p = 0.008^†^
**Presentation characteristics**				
Heart rate (median, IQR, beats/min)	78 (70 - 90)	81.5 (72 - 96)	80 (70 - 92)	p = 0.007*
Systolic blood pressure (median, IQR, mmHg)	130 (110 - 150)	120 (110 - 140)	130 (110 - 140)	p = 0.027*
Cardiac arrest (n, %)	19 (4.4)	12 (5.0)	31 (4.6)	p = 0.722^†^
Creatinine level (median, IQR, mg/dL)	0.94 (0.80 –1.13)	0.79 (0.65 - 1.06)	0.90 (0.74 - 1.11)	p < 0.001*
**Killip classification (n, %)**				p = 0.148^†^
Class I	286 (81.5)	150 (76.9)	436 (79.0)	
Class > I	65 (18.5)	45 (23.1)	110 (20.1)	
**Necrosis marker (median, IQR, ng/ml)**				
High-sensitive cardiac troponin T	3.51 (0.87 - 8.93)	2.50 (0.59 - 6.64)	3.20 (0.77 - 8.20)	p = 0.062*
**ST-segment elevation location (n, %)**				p = 0.645^†^
Anterior	211 (54.7)	108 (49.5)	319 (52.8)	
Inferior	159 (41.2)	94 (43.1)	253 (41.9)	
Lateral	88 (22.8)	59 (27.1)	147 (24.3)	
Posterior	10 (2.6)	6 (2.8)	16 (2.6)	

*IQR: interquartile range; ^†^CAD: coronary artery disease; ^‡^PCI: percutaneous coronary intervention; ^§^CABG: coronary artery bypass grafting. *p-values refers to Mann-Whitney test. ^†^p-values refers to Pearson´s chi-square test.^‡^ p-values refers to Exact Fisher´s test.

### Prehospital conditions and variables

Before admission to the tertiary institution, most of the patients had received care in a general emergency hospital, of which 61.4% were primarily treated within three hours of chest pain onset. Meanwhile, 45.9% were referred to tertiary care after five hours of symptom onset, and 24% after more than ten hours. Nevertheless, when symptoms onset to hospital care (pain-to-door time), to ECG acquisition (pain-to-ECG time), and from tertiary hospital admission to PCI (door-to-balloon time) were evaluated, it was found that these timings were not significantly different according to sex. However, we found a different total ischemia time (symptoms onset to balloon time), showing that men underwent primary PCI more quickly than women (Table 2).

**Table 2:** – Sex differences and similarities regarding clinical and invasive treatment

Variable	Male	Female	All	p Value

	n (%)	n (%)	n (%)	
**Clinical scenario**				
Total ischemia time (median, IQR*, hours)	13.86 (8.81 - 17.00)	18.00 (10.5 - 20.33)	14.77 (9.30 - 19.68)	p<0.001*
**Pain-to-door time (hours)**				p=0.416*
Until 2	64 (17.6)	28 (13.7)	92 (16.2)	
2–5	100 (27.5)	50 (24.5)	150 (26.5)	
5–10	96 (26.4)	59 (28.9)	155 (27.3)	
> 10	103 (28.4)	67 (32.8)	170 (30)	
**Pain-to-ECG† time (hours)**				p=0.265*
< 1	117 (37.4)	45 (29.8)	162 (34.9)	
1–3	92 (29.4)	57 (37.7)	149 (32.1)	
3–10	68 (21.7)	33 (21.9)	101 (21.8)	
> 10	36 (11.5)	16 (10.6)	52 (11.2)	
**Door-to-balloon time (hours)**				p=0.174*
< 1	59 (31.1)	27 (23.1)	86 (28.0)	
1–4	58 (30.5)	33 (28.2)	91 (29.6)	
4–10	42 (22.1)	27 (23.1)	69 (22.5)	
> 10	31 (16.3)	30 (25.6)	61 (19.9)	
Fibrinolytic therapy	17 (4.0)	6 (2.5)	23 (3.4)	p=0.304^†^
IIb/IIIa glycoprotein inhibitor use	27 (5.9)	14 (5.5)	41 (5.8)	p=0.802^†^
Spontaneous MI^‡^	372 (96.1)	209 (97.7)	581 (96.7)	p=0.836^‡^
Stent thrombosis-related MI^‡^	10 (2.6)	3 (1.4)	13 (2.2)	p=0.836^‡^
**Cardiac catheterization findings**				p=0.515^†^
Uni-arterial involvement	172 (41.1)	84 (36.5)	256 (39.5)	
Bi- or tri-arterial involvement	104 (54.1)	130 (56.5)	356 (55.0)	
Left main disease involvement	24 (5.8)	14 (6.3)	38 (6.0)	
No significant obstruction	20 (4.8)	16 (7.0)	36 (5.6)	
**PCI^**§**^ details**				
Radial access	155 (46.1)	45 (23.7)	200 (38)	p<0.001^‡^
1 or 2 implanted stents	309 (91.4)	176 (91.2)	485 (91.3)	p=0.996^†^
Bare-metal stent implanted	302 (92.9)	172 (92.0)	474 (92.6)	p=0.849^‡^
Success procedure rate	311 (96.0)	176 (94.1)	487 (95.3)	p=0.336^†^
TIMI^//^ 3 final flow	254 (89.4)	146 (87.4)	400 (88.7)	p=0.671^‡^
Major bleeding complications	5 (1.6)	6 (3.3)	11 (2.2)	p=0.344^‡^
CABG^¶^ indication	9 (2.7)	6 (3.3)	15 (2.9)	p=0.705^†^
In-hospital overall mortality	22 (5.6)	29 (13.2)	51 (8.3)	p=0.001^‡^

*IQR: interquartile range; ^†^ECG: electrocardiogram; ^‡^MI: myocardial infarction; ^§^PCI: percutaneous coronary intervention; ^//^TIMI: thrombolysis in myocardial infarction; ^¶^CABG: coronary artery bypass grafting. *p-values refer to the Mann-Whitney test. ^†^p-values refers to Pearson´s chi-square test. ^‡^p-values refer to Exact Fisher´s test.

### Clinical evaluation and electrocardiogram

The majority of patients had a spontaneous MI. At hospital admission, chest pain, cardiogenic shock, cardiac arrest, Killip class, and the indication for an invasive treatment were not different between women and men. Around one-third of patients had a 12-lead ECG performed within the first hour of symptoms onset, and most individuals had received an ECG diagnosis within three hours, without significant differences between sexes.

Sinus rhythm and anterior wall ST-elevation followed by inferior wall ST-elevation were the most common presentations and similar between sexes, while isolated ST-elevation in augmented vector right (aVR) lead and left-or-right complete-branch block were uncommon.

### Cardiac catheterization and primary PCI

Emergency cardiac catheterization followed by primary PCI was performed in most patients. Some patients underwent elective PCI, and a minority had no indication for an invasive strategy due to non-cardiac clinical complications such as gastric bleeding or frailty, especially in very elderly patients, at the attending physician’s discretion. Non-obstructive CAD was found in 36 patients, irrespective of their sex.

Uni-, bi-, or multi-arterial and left main disease involvement did not differ between the sexes. Radial access was more likely to be performed in men compared to women. Stent number and type (bare metal or drug-eluting), PCI success, and Thrombolysis in Myocardial Infarction (TIMI) final flow were not different between the groups.

The vast majority of patients received a bare-metal stent. Any complication related to PCI occurred in a small number of patients and was mostly due to bleeding. Baseline creatinine levels and systolic blood pressure were lower in women than in men, while heart rate was higher in women than in men.

### In-hospital mortality

Fifty-one patients died. Women had a significantly worse mortality rate, compared with men. The results of the multivariate logistic regression model are presented in Table 3. Age, cardiac arrest at admission, Killip class > I, and the female sex were independent predictors of in-hospital mortality. The final model (Figure 1) was 94.1% accurate (c^2^ = 12.45, p = 0.132) in predicting overall in-hospital mortality with a good model adjustment, and the c-index was 0.85, 95% CI 0.77–0.93.

**Table 3:** – Univariate and multivariate logistic regressions for in-hospital mortality prediction

Variable	Univariate regression		Multivariate regression	
	
	OR* (CI 95%)	p Value	OR* (95% CI^**†**^)	p Value
Age (per 1 year increase)	1.06 (1.04–1.09)	< 0.001	1.06 (1.02–1.09)	0.002
Female sex	2.66 (1.48–4.65)	0.001	2.79 (1.15–6.76)	0.023
Troponin T level	1.03 (0.99–1.07)	0.185	1.05 (0.99–1.11)	0.143
Initial creatinine level	1.17 (1.00–1.14)	0.047	1.48 (0.96–2.20)	0.079
Heart rate/pulse	1.02 (1.00–1.04)	0.002	1.02 (1.00–1.04)	0.055
Systolic blood pressure	0.97 (0.96–0.98)	< 0.001	0.97 (0.96–0.99)	0.001
Cardiac arrest at admission	10.45 (4.10–26.58)	< 0.001	6.77 (1.25–36.63)	0.027
Killip class > I	4.74 (2.58–8.71)	< 0.001	3.09 (1.22–7.81)	0.017

The final logistic model was statically significant and the Hosmer-Lemeshow chi-square statistic (a measure of the goodness-of-fit) was c^2^ = 12.45 and had a p value of 0.132. ^*^OR: odds ratio; ^†^CI: confidence interval.

**Figure 1:** – Final multivariate model for in-hospital mortality after ST-segment elevation myocardial infarction.

## Discussion

As far as we are concerned, our study is the largest cohort proposed specifically to evaluate sex differences after STEMI in Brazil. It showed that the female sex remained at higher risk for in-hospital mortality after STEMI in contrast to GRACE ACS risk score, in which sex is not a predictor. Despite that women were older than men in our study, the female sex remained associated with impaired survival even after multivariable adjustment (Central Illustration), which included age in line with de-Miguel-Balsa E et al. study findings. When validating GRACE, they found the female sex as an independent predictor of hospital mortality in the STEMI subpopulation, and sex did not substantially improve the discriminative ability of the GRACE score.^16^

Furthermore, although women displayed a higher prevalence of comorbidities, such as hypertension, diabetes, and dyslipidemia, the GRACE ACS does not account for these in the prediction of mortality after ACS,^11,16^ even though the Charlson comorbidity index was similar in both sexes.^17^ In addition, other important cardiovascular risk factors (smoking, CAD family history, prior stroke or MI, peripheral artery disease, and chronic kidney disease) that have a greater demonstrated impact on higher mortality were not significantly different between men and women.^4,6^

Further, it has been suggested that atypical symptoms could delay ACS treatment and lead to less invasive management in women compared to men.^18^ In line with the different total ischemia time found in our study, a longer waiting period for primary PCI is unfavorable for women. Even though it was not part of the GRACE mortality prediction model, it may have contributed to higher mortality among women in this study. However, we did not find significant differences in the pain-to-door, pain-to-ECG, and pain-to-balloon times or the indication to PCI rate between sexes.

Regarding access to cardiac catheterization and PCI procedures, our study showed a significant difference in artery access choice, with radial access being less frequent in women. Higher mortality rates may be related to increased use of femoral access for ACS PCI among women due to major bleeding.^19,20^ Yet, bleeding complications were uncommon in our study, and we did not find any difference based on sex. Further, anterior STEMI was the most common presentation in our study cohort and is generally associated with worse outcomes compared with other ACS presentations. Nonetheless, MI location was not different between men and women.

Furthermore, although primary PCI was indicated for 85.6% of patients, a considerable number of patients presenting with non-obstructive CAD, indication for coronary artery bypass surgery (CABG), and a subacute phase of STEMI had PCI contraindicated, irrespective of their sex.

It is widely recognized that women are less frequently diagnosed and treated after ACS.^18,21^ However, this was not demonstrated in our study, as women and men had similar drug prescriptions, PCI indications, and success rates. Although the percentage of bare metal stents was high when comparing current reports, it was irrespective of sex and reflected the limited financing for the national public health system.

### Mortality and a multivariate model

The higher rate of in-hospital mortality observed in women is in line with previous reports.^1,2^ This finding has been attributed to older age and higher burden of comorbidities of women. However, several studies showed increased mortality in women even after adjustment for these confounders.^22-26^ Therefore, after adding the female sex to our model, the GRACE ACS score established that variables such as age, cardiac arrest at admission, and Killip class > I have remained independent in-hospital mortality predictors. On the other hand, abnormal troponin, creatinine level, and heart rate were excluded from the model.

### Limitations

Limitations to this study relate to the observational design. We cannot rule out selection bias and all residual and/or unmeasured confounding. Moreover, the sample is derived from a single-center experience analyzing only in-hospital mortality, which may underestimate the prevalence of some cardiovascular risk factors, and misclassification of sex and diagnosis may have occurred. For this analysis, only hospitalized patients were considered, and the possibility of having higher mortality rates after STEMI in men than women before hospital admission may have been a selection bias. Regarding measurements related to the possible influence of sex in the treatment, we believe that by including all consecutive patients and performing in-hospital data collection after standard STEMI treatment, we minimized this bias, although it was not excluded. Moreover, the limited statistical power of the study may have affected the variable selection in our multivariate model.

### Implications for practice and/or policy

This finding highlights the outcome disparities after ACS in women, compared with men, and should be a prominent factor in the implementation of health policies to minimize women’s mortality following ACS. Clinical practitioners should be constantly trained to observe sex-related particularities in clinical scenarios, such as symptom descriptions, risk factors, and clinical evolution after STEMI.

## Conclusions

In conclusion, the female sex, in addition to the GRACE score, remained associated with a higher risk for in-hospital mortality after STEMI. Besides, treatment guidelines that are recommended and followed for all STEMI patients in this study may not be appropriate for women. Still, the female sex should be reconsidered in future mortality risk score iterations.
